# Unexpected benefits of and lessons learned from shifting to virtual focus group discussions in the BEECON trial

**DOI:** 10.1186/s13104-022-05950-3

**Published:** 2022-03-04

**Authors:** Helen Lindau, Francisco Ramos-Gomez, Jeremiah Garza, Tracy Finlayson, Morgan Pareja, Jenny Liu, Stuart Gansky

**Affiliations:** 1 University of California, 10833 LeConte Ave, Los Angeles, CA 90026 USA; 2 Los Angeles, CA USA; 3 San Diego State University, 9245 Sky Park Court, San Diego, CA 92123 USA; 4 Department of Social and Behavioral Sciences, University of California, San Francisco, Institute for Health and Aging, 490 Illinois St, San Francisco, CA 123J94158 USA; 5 Center to Address Disparities in Children’s Oral Health (Known as CAN DO), School of Dentistry, Department of Preventive & Restorative Dental Science, University of California, P.O. Box #1361, San Francisco, CA 94143 USA

**Keywords:** Qualitative data, Oral health, Focus groups, Virtual data collection

## Abstract

**Objective:**

The COVID-19 pandemic has forced many human subjects research to halt in-person activities and pivot to virtual engagement, including Focus Groups (FGs). We highlight learnings from our experience of hosting virtual FGs from our BEhavioral EConomics for Oral health iNnovation (BEECON) study focusing on oral hygiene behaviors among low-income, predominantly Hispanic families, including practical tips and potential pitfalls to avoid for researchers considering virtual engagement.

**Results:**

There can be particular benefits to holding virtual sessions among minority parents of young children—to provide flexibility, comfort, and reduced logistical barriers for participation—while still facilitating friendly conversation with minimal distractions. However, extensive preparation is needed to ensure smooth execution and minimal distractions.

## Introduction

The COVID-19 pandemic has forced many human subjects research to halt in-person activities and pivot to virtual engagement, including for Focus Groups (FGs). While virtual FGs have been reported as feasible [1–3], in-person FGs are the norm. Virtual FGs may offer anonymity, convenience, cost-savings, and potentially comparable data quality to in-person FGs. Yet, digitally reaching vulnerable populations and adhering to research protocols can be challenging, depending on participants’ access to broadband and digital devices, familiarity with their functionality, and the nature of the research content [4]. Our recent experience conducting FGs with low-income, predominantly Latino families in Los Angeles, CA, showed that engaging virtually was not only feasible, but also offered several advantages including enhancing the participant experience. Here, we reflect on lessons learned, sharing some practical tips and potential pitfalls to avoid when implementing virtual FGs.

## Main text

### Methods

#### Setting and participants

The BEhavioral EConomics for Oral health iNnovation (BEECON) trial aims to improve oral hygiene behaviors among low-income, predominantly Hispanic families and their children in Early Head Start (EHS) and other daycare programs in Los Angeles, CA, with financial incentives (NCT03576326). FGs aimed to explore participants’ attitudes and perceptions of the intervention [5]. The first phase of qualitative research involving five FGs and seven key-informant interviews was conducted in-person in 2019 [6]. The final two FGs with only intervention arm participants were to occur in November 2020^1^ but COVID-19 public health restrictions precluded in-person engagement. We pivoted to holding two virtual FGs (one in Spanish, one in English) over Zoom. Intervention participants with both high and low levels of toothbrushing performance were invited to elicit a range of experiences and perspectives. We descriptively compare the characteristics of our prior in-person focus group participants to those convened virtually in terms of their demographics, technology-readiness scale scores, and distance of their residence to the dental clinic.

#### Data collection

**Fig. 1:**
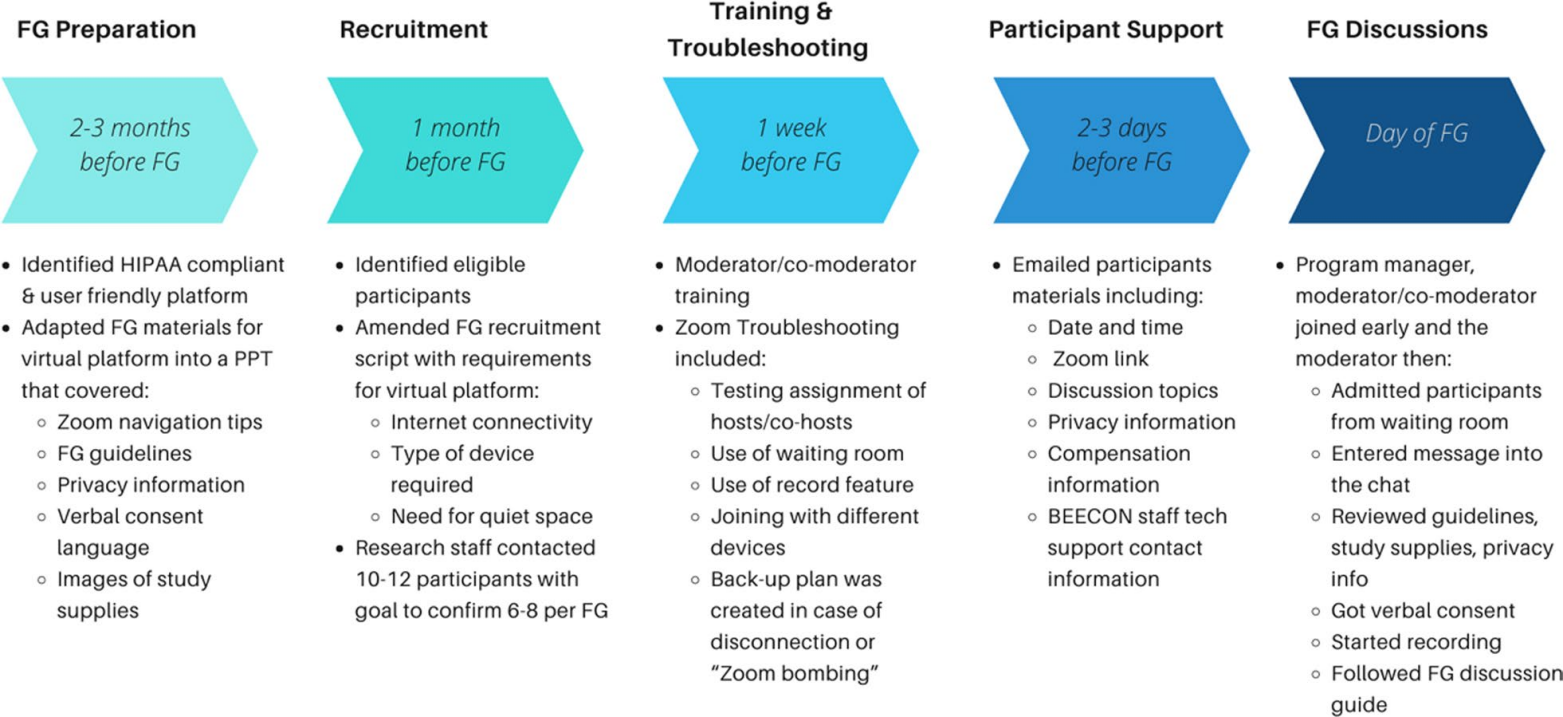
Timeline of planning and preparation for virtual FGs

We began planning three months in advance (Fig. 1). Institutional Review Board (IRB) protocols were amended to accommodate virtual data collection (UCSF 17-23786).

#### Identifying a suitable platform

Of the handful of platforms that were reviewed, Zoom was the only option that our team could identify that was institution-supported and HIPAA- (Health Information Portability and Accountability) compliant. Research team members also recently had positive experiences using Zoom for virtual data collection in another study, and many participants were familiar with it from their children’s distance learning.

#### Adjusting procedures

We kept the FG size (6–10 participants) and duration (90 min) the same as previous in-person sessions. We adjusted two aspects to allow sufficient time for interactive discussion: (1) added verbal consent for audio-recording the session,^2^ and (2) rather than giving a short questionnaire^3^ at the end of the discussion, we administered it via telephone the day before (see *Preparing participants*). Childcare accommodations previously required during in-person FGs with parents were unnecessary.

#### Adapting tools

We added questions about COVID-19 impacts to the discussion guide. During the activity introduction, the moderator shared a PowerPoint with tips and screenshots for how to navigate Zoom functions on different devices,^4^ and explained participation guidelines, privacy procedures, and verbal consent. The moderator displayed images of study materials^5^ and intervention supplies (e.g., toothpaste, toothbrush) when these topics arose during the FG. All materials were translated into Spanish.

#### Recruiting participants

One month before the sessions, we identified a pool of potential participants meeting sampling criteria and recruited them over three weeks. We amended the recruitment script with guidelines for attending virtually—internet connectivity, type of device required, need for a quiet space—and compensation expectations. We contacted 10–12 participants, expecting to confirm 6–8 per session—the same target as prior in-person FGs.

#### Training moderators

Two weeks ahead of time, we trained the moderators on changes to the facilitation materials. The moderators were the same seasoned bilingual and bicultural facilitators of prior in-person FGs. One week ahead, we tested facilitation procedures on Zoom, and refined processes for assigning alternate hosts,^6^ managing the waiting room, recording video, audio, and chats, and connecting from different devices (e.g. laptops, phones). We also prepared a back-up plan with an alternate meeting link and draft email message to participants in case the session was disrupted. We sent a consolidated Live Session Plan with all materials and attendee list to the moderators before each session.^7^ Within two days after each session, the moderators shared their field notes and debriefed with a research team member.

#### Preparing participants

We contacted confirmed attendees three times before the session. We emailed them 2–3 days beforehand to confirm the session time, provide connection information, outline the discussion topics, and explain procedures for recording, voluntary participation, compensation, privacy safeguards, and technical assistance. The day before, we called each participant to review preparations, administer the short questionnaire, and answer questions. On the morning of, we sent a reminder text message. As with our in-person FGs, virtual sessions were held on weekday evenings.

#### Recording the FG

Because Zoom functionality at the time automatically recorded audio and video upon session initiation and before consent for recording could be obtained, this recording was deleted. Two separate IRB-compliant audio recordings on computer and iPhone were initiated after all participants verbally consented; the duplicate recording was destroyed after one copy was securely saved.

#### Compensating participants

FG participants were compensated with $30 (same amount as in-person FGs) e-gift cards within 2 days.^8^

## Results

### Participation

Of 21 eligible participants contacted, four declined because of scheduling conflicts or were not reached. None declined due to virtual engagement requirements (i.e., internet and device access, quiet space); one declined because they “did not like” virtual meetings. In the reminder call one day before, all participants expressed familiarity with Zoom. We held two virtual FGs with 7 attendees (and one absence) each (Table 1). Recruitment and final attendance was better for the virtual FGs than prior in-person FGs, which had 24 participants across 5 FGs.

**Table 1:** Participant characteristics (mean (std) or percent) by Focus Group

Participant characteristics	In-person (N = 24)	Virtual (N = 14)
Focus group sessions	5	2
Participants	24	14
Mean	4.8	7
Range	2–6	7–7
Demographics		
Age	37.9 (10.9)	34.6 (5.8)
Gender		
Female	91.7	100
Male	8.3	0
Ethnicity		
Hispanic or Latino	70.8	78.6
Not Hispanic/Latino	29.2	14.3
Unknown or Not Reported	0	7.1
Race		
White	8.3	50.0
Black or African American	12.5	0.0
Other Race*	8.3	7.1
Unknown or Not Reported	70.8	42.9
Tech Readiness (TR) Scale	3.5 (0.5)	3.6 (0.6)
Distance they live from clinic (in miles)	3.0 (3.6)	4.9 (2.6)

*Other Race includes Native Hawaiian/Other Pacific Islander, American Indian/Alaska Native, Asian, Other Single Race and More Than One Race

Other attendee characteristics were comparable between prior in-person and virtual FGs. Although all virtual FG attendees were female, this pattern was not unusual given that mothers were the primary caregiver for 95% of study children. Notably, virtual FG attendees lived farther from the clinic compared to prior in-person attendees (mean 4.9 vs. 3 miles, respectively). Attendees of both formats scored similarly on the technology readiness scale.^9^

### Hosting the virtual FG discussion

Moderators launched sessions 15 min before the 6:00 pm start to assign alternate hosts; all attendees joined within two minutes of the hour unlike several instances of very late arrivals during in-person FGs. About half joined on a phone with others joining by tablet or computer. There was no notable difference in quality of participation across device types. All gave verbal consent for audio-recording.

Distractions and disruptions during the sessions were minimal. Nearly all participants effectively navigated the audio and video settings. The moderator muted one attendee due to background noise; none were unexpectedly disconnected. While most engaged on video, individuals participated in other ways: one remained off-video but frequently talked; one responded via chat, which the moderator shared verbally; one did not respond to any questions.

Attendees adhered to the FG guidelines by taking turns, speaking clearly, avoiding side conversations, and muting when not talking or when background noise was present. During the Spanish session, participants raised their own hands (instead of using the icon) and unmuted only when called on. In the English session, participants did not raise hands and spoke at will. Both of these discussion flows were common during prior in-person FGs, and did not seem to affect the quality of the discussion.

All but two participants, who were helped by the moderator, were able to rename themselves in the participant screen. We asked attendees to only use first- or nicknames to preserve confidentiality; it also helped participants to refer to each other.

### Participant receptivity

During session debriefs, moderators noted that attendees seemed relaxed in their homes, and even engaged while multi-tasking (i.e., laundry, breastfeeding). Participants specifically noted relief about not having to travel. For some situated in a shared space, participants facilitated their own privacy; about one-third wore headphones, and one turned off her camera whenever another person was present.

## Discussion of lessons learned

Despite our concerns that our low-income study population may face challenges with technology, our virtual FGs resulted in higher-than-anticipated attendance compared to our prior in-person FGs. Virtual FGs may have even improved participants’ experience of the activity compared to in-person engagement. It helped overcome logistical challenges for both study staff and attendees, and reduced meeting costs (for providing childcare, refreshments, and transportation). Importantly, it enabled those living farther away to participate. Compared to in-person FGs, participants were also generally more comfortable during the virtual FGs; discussions were relaxed and the tone was warm and friendly. The chat box also provided one attendee an additional way to participate in a way that was comfortable for her.

Our experience suggests that virtual engagement was feasible given that study participants were already digitally connected. While 89% of American households have a computer with internet access [8], internet use is also generally high among Hispanic adults (78% in 2012) [9], and prior studies with our trial demographic report common use with video interfaces (e.g., FaceTime, Skype) [4, 10]. The study Community Advisory Board and EHS staff noted that many parents regularly used digital technologies, and according to our formative research, 50% of caregivers had used instant or video messaging in the past week [5]. The BEECON intervention itself also involved using tech-enabled devices (e.g., Bluetooth electric toothbrush, text messages, toothbrushing app), so it is possible that our sample was already “tech-savvy.”

Nonetheless, adapting study procedures and preparing for virtual FGs was highly involved and necessitated a long lead time to accommodate our study participants’ specific needs. Our preparations helped to ensure smooth engagement without technical distractions. Additionally, having the same moderators as prior in-person FGs was an asset as they were familiar with the study and the FG guide, were able to contribute edits, anticipate problem areas, and had experience facilitating Zoom group discussions from other studies.

## Conclusion

 Virtual FGs offer a promising alternative to in-person qualitative data collection, the success of which has encouraged our other research teams to similarly move to a virtual format using our experience as guidance for their extensive preparations. There can be particular benefits to holding virtual sessions among minority parents of young children—to provide flexibility, comfort, and reduced logistical barriers for participation—while still facilitating friendly conversation with minimal distractions.

## Limitations

Our study was not explicitly designed to assess the feasibility and acceptability of holding virtual focus groups compared to in-person discussions. As such, we did not ask participants about acceptability directly, but rather rely on notes and the experiences of the discussion moderators, as well as process outcomes. Retrospective comparison with in-person discussion relied on recall from study staff, supplemented with participant attendance data. Additionally, we only assessed one virtual platform during these focus groups.

## Data Availability

Data sharing is not applicable to this article as no datasets were generated or analyzed during the current study.

## Abbreviations

FG(s): Focus group(s)
BEECON: Behavioral economics for oral health innovation
EHS: Early head Start
HIPAA: Health information portability and accountability
IRB: Institutional review board

## Footnotes

1 after 6 months of intervention exposure and an additional 6 months of post-intervention toothbrushing data collection.

2 Written signed consent was completed for the main trial which included description of the FG activities that participants would have an opportunity to participate in.

3 The 10 min, 28-item questionnaire included a technology-readiness scale [7] questions about opportunity cost and intrinsic motivation, use of the study equipment (electric toothbrush, toothpaste pump, Sonicare 4 Kids app), and receipt of text messages and incentives earned during the intervention.

4 Changing display name, turning audio and video on/off.

5 Study flyer, example text message reminders, Sonicare 4 Kids mobile app.

6 Due to organizational IT restrictions, alternative hosts could not be assigned at the time of scheduling, necessitating the original host to start the Zoom session and assign alternative hosts before leaving.

7 The Session Plan included the following information: Zoom connection information, attendance roster (participant name, email address) with background trial information (outcomes, questionnaire completion status), staff roles and contact information, session format and flow (e.g., arrival, introductions, discussion, wrap-up), prepared welcome messages for the chat box, link to secure server location where recordings should be saved, a “backup” email to participants in the event the session was disconnected, FG discussion guide and associated PowerPoint presentation.

8 E-gift cards were similarly offered in our main trial when pandemic-related restrictions were imposed and had been well-received among study participants.

9 The overall TR score for each participant was obtained by averaging the scores of the four dimensions (Optimism, Innovativeness, Discomfort, Insecurity) which were scored using a five-point scale (1- strongly disagree to 5- strongly agree).
